# Modulation of stress-related behaviour by preproglucagon neurons and hypothalamic projections to the nucleus of the solitary tract

**DOI:** 10.1016/j.molmet.2024.102076

**Published:** 2024-11-25

**Authors:** Marie K. Holt, Natalia Valderrama, Maria J. Polanco, Imogen Hayter, Ellena G. Badenoch, Stefan Trapp, Linda Rinaman

**Affiliations:** 1 Florida State University, Department of Psychology and Program in Neuroscience, Tallahassee, FL, USA; 2 Centre for Cardiovascular and Metabolic Neuroscience, Department of Neuroscience, Physiology & Pharmacology, UCL, London, UK; 3 University of Warwick, School of Life Sciences, Coventry, UK; 4 GlaxoSmithKline Pharmaceuticals, London, UK

**Keywords:** Glucagon-like peptide-1, Acute stress, Nucleus of the solitary tract, Corticotropin releasing hormone, Appetite, Anxiety-like behaviour

## Abstract

Stress-induced behaviours are driven by complex neural circuits and some neuronal populations concurrently modulate diverse behavioural and physiological responses to stress. Glucagon-like peptide-1 (GLP-1)-producing preproglucagon (PPG) neurons within the lower brainstem caudal nucleus of the solitary tract (cNTS) are particularly sensitive to stressful stimuli and are implicated in multiple physiological and behavioural responses to interoceptive and psychogenic threats. However, the afferent inputs driving stress-induced activation of PPG neurons are largely unknown, and the role of PPG neurons in anxiety-like behaviour is controversial. Through chemogenetic manipulations we reveal that cNTS PPG neurons have the ability to moderately increase anxiety-like behaviours in mice in a sex-dependent manner. Using an intersectional approach, we show that input from the paraventricular nucleus of the hypothalamus (PVN) drives activation of both the cNTS as a whole and PPG neurons in particular in response to acute restraint stress, but that while this input is rich in corticotropin-releasing hormone (CRH), PPG neurons do not express significant levels of receptors for CRH and are not activated following lateral ventricle delivery of CRH. Finally, we demonstrate that cNTS-projecting PVN neurons are necessary for the ability of restraint stress to suppress food intake in male mice. Our findings reveal sex differences in behavioural responses to PPG neural activation and highlight a hypothalamic-brainstem pathway in stress-induced hypophagia.

## Highlights

• Female mice suppress feeding more robustly after cNTS PPG neuron activation.
• cNTS PPG neuron activation increases anxiety-like behaviour; sex-dependent effects.
• A CRH-rich PVN input drives stress-induced activation of cNTS and PPG neurons.
• The cNTS receives stress-activated input from multiple CRH-expressing regions.
• CRH activates the NTS; CRH receptors are likely presynaptically expressed.

## 1 Introduction

Behavioural and physiological responses to stressors are essential for survival and are tightly controlled by the brain. Exposure to stressful stimuli leads to activation of the hypothalamic-pituitary-adrenal (HPA) axis, sympathetic arousal, and elicitation of adaptive behaviours, including heightened vigilance and decreased exploration and food intake [[1], [2], [3]]. While hypothalamic areas are often considered the primary site of stress processing and modulation [4], multiple brainstem regions are essential for the integration of signals elicited by both interoceptive and psychogenic stressors [2,5]. The caudal part of the nucleus of the solitary tract (cNTS) is particularly well-positioned to integrate such signals [[6], [7], [8], [9]]. Neurons within the cNTS are sensitive to both interoceptive and psychogenic stressors [8], presumably mediated by their receipt of interoceptive sensory input from the spinal cord and vagal afferents along with descending input from higher brainstem regions, cerebral cortex, limbic system, and hypothalamic nuclei, including the paraventricular nucleus of the hypothalamus (PVN) [7,[10], [11], [12], [13]].

Acute psychogenic stressors elicit robust suppression in feeding which can last hours to days in rats [14,15]. Circuits driving this stress-induced hypophagia partially overlap with satiation circuits [14] that include neurons within the cNTS. Multiple cell types within the cNTS have the ability to modulate feeding [[16], [17], [18], [19], [20]], including preproglucagon (PPG)-expressing neurons that synthesize glucagon-like peptide-1 (GLP-1). Indeed, chemogenetic and optogenetic activation of cNTS^PPG^ neurons robustly suppresses food intake in mice [[21], [22], [23], [24], [25]] and in rats [26]. In mice, while cNTS^PPG^ neurons are necessary for limiting intake of large meals [21,24], interfering with endogenous central GLP-1 receptor signalling either pharmacologically, genetically, or through inhibition of cNTS^PPG^ neuron activity has limited impact on *ad libitum* chow feeding [21,22,24,25,[27], [28], [29]]. Evidence suggests that rather than contributing to meal-induced satiation under normal physiological conditions, cNTS^PPG^ neurons are more strongly engaged by stressful conditions that inhibit food intake. Indeed, cNTS^PPG^ neurons are activated by a variety of acute stressors that suppress food intake in both rats and mice [11,28,30,31], and GLP-1 acts centrally to generate stressor-like physiological responses that include activation of the HPA axis [32,33] and increased heart rate [34,35]. Additional evidence indicates that cNTS^PPG^ neurons are necessary for stress-induced hypophagia in mice [24], that stress-induced hypophagia is significantly reduced in rats after central blockade of GLP-1 receptors [30,36], and that central endogenous GLP-1 contributes to anxiety-like behaviours in rats [30,33,36,37]. Surprisingly, however, a previous study using chemogenetic activation of cNTS^PPG^ neurons in mice failed to detect any changes in anxiety-like behaviour assessed in the elevated plus maze or open field [23], although only male mice were included in that study (R. Gaykema, personal communication).

The current study had two objectives: first, to reassess the role of cNTS^PPG^ neurons in anxiety-like behaviours in male and female mice tested in the open field and to additionally test the role of these neurons in acoustic startle responses, and second, to identify inputs from the PVN and other brain regions that potentially drive stress-induced activation of cNTS^PPG^ neurons. We previously reported that cNTS-projecting PVN neurons are activated in response to acute restraint stress in mice [10], but the role of this pathway in activating cNTS^PPG^ neurons and driving behavioural responses to stress has not been tested.

## 2 Materials and methods

Experiments carried out in the US (Experiments 1–3,5) were approved by the Florida State University (FSU) Institutional Animal Care and Use Committee, and were consistent with the US Public Health Service's Policy on the Humane Care and Use of Laboratory Animals and the NIH Guide for the Care and Use of Laboratory Animals. Experiment 4 was carried out in the UK and received ethical approval by the Home Office and was performed in accordance with the U.K. Animals (Scientiﬁc Procedures) Act, 1986.

### 2.1 Animals

Male and female mGlu-Cre/tdRFP (n = 38), mGlu-Venus (N = 15), and CRH-ires-Cre (N = 8, JAX stock #012704) [38,39] transgenic mice were bred in house; wildtype, male C57BL/6J mice (JAX stock #000664; N = 13M) were obtained from The Jackson Laboratories. Details regarding viral injections, sample sizes, and animal ages and sex are provided in Table 1.

**Table 1:** Experimental details.

Experiment	Mouse strain	Viral transduction, sample size and sex	Age at surgery (months±SD)	Age at perfusion (months±SD)
1) Chemogenetic activation of PPG neurons	Glu-Cre/tdRFP	hM3Dq (N = 8F,6M) mCherry (N = 8F,6M)	3.0 ± 1.4	8.87 ± 2.2
2) Monosynaptic retrograde tracing from PPG neurons	Glu-Cre/tdRFP	RABV (N = 5F,3M)	3.5 ± 0.12	3.7 ± 0.12
3) Chemogenetic inhibition of NTS-projecting PVN neurons	C57BL/6J	hM4Di (N = 5M) GFP (N = 8M)	2.9 ± 0.05	5.4 ± 0.03
4) Mapping of CRH input to the NTS	CRH-ires-Cre	tdTomato (N = 3F,5M)	2.0 ± 0.41	3.3 ± 0.56
5) Neural activation in response to central exogenous CRH	mGlu-Venus	Cannulation of lateral ventricle (N = 6F,6M)	4.9 ± 0.84	5.6 ± 0.83

Mice were individually housed from the start of behavioural experiments and were kept on a 12 h light/dark cycle. They had *ad libitum* access to water and rodent chow (Purina) unless otherwise stated and were habituated to handling and injection procedures every day for at least one week prior to experimentation. mGlu-Venus mice [40] express a yellow ﬂuorescent protein reporter, Venus, under the control of the glucagon promoter, allowing visualization of PPG neurons [41]. mGlu-Cre transgenic mice express Cre recombinase under the control of the glucagon promoter, allowing selective targeting of GLP-1-expressing PPG neurons [24,42,43]. These cells also express the fluorescent reporter, tdRFP, in a Cre-conditional manner [44]. Local colonies of mGlu-Cre/tdRFP transgenic mice were established at FSU in 2013 from a strain received from Frank Reimann at Cambridge University (UK). The original Cambridge mGlu-Cre mice were generated in 2008 and maintained for >20 generations before receipt by FSU. At FSU, mGlu-Cre mice have been maintained for >15 generations on a C57BL/6 background.

### 2.2 Exp 1: chemogenetic activation of cNTS^PPG^ neurons

#### 2.2.1 Stereotaxic injections

mGlu-Cre/tdRFP mice were anaesthetized using isoflurane (1–3%, 1.5 ml/min in O_2_) and placed in a stereotaxic frame with the nose pointing downwards to expose the dorsal surface of the neck and facilitate access to the caudal brainstem. An incision was made through the skin along the midline extending from the occipital crest to the first vertebra and the underlying muscles were separated to expose the roof of the fourth ventricle caudal to the cerebellum. The meningeal layer was penetrated using a 30g needle and obex was visualized. To target the cNTS with AAVs (Table 2), the tip of a glass needle was inserted 400 μm lateral and 100 μm rostral to obex, and then lowered 350 μm below the dorsal surface of the brainstem.

**Table 2:** Viral titers, injection volumes, and sources.

Virus	Titer (pfu/ml)	Volume	Source	Reference
AAV8-hSyn-DIO-mCherry	2.1 × 10^12^	250 nl	Addgene #44361-AAV8	[77]
AAV8-hSyn-DIO-hM3Dq:mCherry	2.2 × 10^12^	250 nl	Addgene #50459-AAV8	[78]
AAV8-hSyn-DIO-hM4Di:mCherry	2.0 × 10^12^	400 nl	Addgene #44362-AAV8	[77]
AAV1-CAG-FLEX-EGFP	2.0 × 10^12^	400 nl	Addgene #51502-AAV1	[79]
AAVrg-hSyn-Cre	1.2 × 10^12^	200 nl	Addgene #105553-AAVrg	Gift from James M. Wilson
AAVrg-CAG-FLEX-tdTomato	2.6 × 10^13^	200 nl	Addgene # 28306-AAVretro	Gift from Edward Boyden
AAV5-EF1a-FLEX-TVA:mCherry	2.13 × 10^12^	100 nl	GVC-AAV-67, Stanford gene vector and virus Core	[80]
AAV8/733-CAG-FLEX-RabiesG	2.4 × 10^13^	100 nl	GVC-AAV-59, Stanford gene vector and virus Core	[80]
(EnvA)-RABV-ΔG-GFP	2 × 10^8^	400 nl	Kevin Beier, University of California at Irvine, CA	[81]

#### 2.2.2 Food intake measurements

A minimum of two weeks after stereotaxic injection, mice were individually housed in cages fitted with the BioDAQ food-intake monitoring system (Research Diets, New Brunswick, New Jersey) and left to habituate to the feeding system and handling for at least one week prior to experimentation. On test days, chow access gates were closed to prevent feeding for 3 h prior to dark onset. Using a mixed-model design with a minimum of 48 h between conditions, mice were injected 30 min s before dark onset with either saline vehicle (control) or vehicle containing clozapine-*N-oxide* dihydrochloride [CNO; Tocris; 2 mg/kg, 2 ml/kg, i.p.; dose based on previous studies [21,23,24,35]. At dark onset, food gates were opened to provide chow access, with intake measured continuously by the BioDAQ. Meal-pattern data were subsequently extracted using the BioDAQ Data Viewer (Research Diets), with a meal defined as a feeding episode in which at least 0.02g of chow was consumed with an intermeal interval of at least 300s. Cumulative food intake, average meal size, and average meal duration were analysed during the first 6 h after dark onset, as well as latency to begin the first meal and first meal size.

#### 2.2.3 Acoustic startle response

Using a mixed-model design with virus as a between-subjects factor and treatment (CNO vs saline) as within-subjects factor, mice were tested in the SR-Lab-Startle Response System (San Diego Instruments, San Diego, CA) during the light phase of the photoperiod (2–10 h after light onset). Each mouse was tested under both CNO and saline conditions using a randomised, counterbalanced design with at least 48 h between tests. On each day of testing, mice were moved to the test room and left to acclimatize for 30 min after which they were injected with CNO (2 mg/kg, 2 ml/kg) or saline (2 ml/kg). Thirty mins after injection, mice were placed individually into a clear acrylic enclosure which allowed mice to turn around without constraint (San Diego Instruments, San Diego, CA). Mice were left to acclimatize for 5 min in the darkened startle chamber with a constant background noise level of 50 db. Following this initial period, mice were exposed to a series of 50 ms white noise bursts at 75, 90, and 105 db (10 repeats of each) in a randomly generated order with random intervals of 20–40 s between noise bursts. Testing lasted 23 min in total. All testing was done during the light cycle.

#### 2.2.4 Open field test

Using a between-subjects design (with virus as the between-subjects factor), mice were injected with CNO (2 mg/kg, 2 ml/kg) 30 min prior to individual testing in a novel open field (50 cm × 50 cm), with exploratory behaviour recorded for 20 min s during the light phase (5–9 h after light onset) of the photoperiod. Location was tracked using the ezTrack Location Tracker open-source software [45]. Total distance travelled and time spent in the central region (30 × 30 cm) of the open field were analysed.

#### 2.2.5 Terminal procedure

Mice were injected with either saline (2 ml/kg) or CNO (2 mg/kg, 2 ml/kg) during the light phase and were transcardially perfused 90 min later as described in section 2.7.1.

### 2.3 Exp 2: rabies virus-mediated circuit tracing

#### 2.3.1 Stereotaxic injections targeting cNTS

Using the surgical approach described in section 2.2.1, mGlu-Cre/tdRFP mice (n = 4M, 4F) received cNTS microinjection of two helper AAVs encoding rabies glycoprotein (G) and TVA receptor (Table 2). Three weeks later, the same mice were moved into a Biosafety Level 2 (BSL2) laboratory where they received cNTS-targeted microinjection of EnvA-pseudotyped G-deleted rabies [(EnvA)-RABV-ΔG-GFP; Table 2]. As previously described [10], (EnvA)-RABV-ΔG-GFP was injected at each of two medial-lateral injection sites relative to obex: (1) 250 μm lateral, 100 μm rostral, and 450-350 μm below the surface of the brainstem; and (2) 400 μm lateral, 100 μm rostral, and 450-350 μm below the surface of the brainstem. Viral titers, injection volumes, and sources are listed in Table 2. Rabies virus-injected mice remained housed in the BSL2 laboratory until their terminal procedure.

#### 2.3.2 Terminal procedure

One week after injection of rabies virus, mice were restrained in a decapicone for 30 min s (N = 2F,1M) or left undisturbed (non-handled; N = 3F, 2M) in their home cages. Ninety minutes after the onset of restraint stress, or at a comparable time for controls, mice were anaesthetized and transcardially perfused as in section 2.7.1. Brains were extracted and processed as described in section 2.7.2.

### 2.4 Exp 3: chemogenetic inhibition of NTS-projecting PVN neurons

#### 2.4.1 Stereotaxic injections targeting cNTS and PVN

Wildtype, male C57BL/6J mice (N = 13) were anaesthetised using isoflurane and placed in a stereotaxic frame. The cNTS was targeted as described above (section 2.2.1) for bilateral microinjection of AAVrg-hSyn-Cre (Table 2) to induce Cre expression in cNTS-projecting neurons. Following cNTS injection in the same surgical session, the skull was levelled, and a skin incision was made along the midline of the skull overlying the diencephalon. A single hole was drilled in the skull to allow bilateral targeting of the PVN with AAV8-hSyn-DIO-hM4Di:mCherry or AAV1-CAG-FLEX-EGFP (Table 2) at the following coordinates from bregma: 820 μm caudal, 100 μm lateral, and 4.75 mm ventral.

#### 2.4.2 Food intake experiments

To assess the effect of chemogenetic inhibition of cNTS-projecting PVN neurons on baseline food intake, chow access was prevented for 3 h prior to dark onset, and CNO (2 mg/kg, 5 ml/kg) or saline (5 ml/kg) was injected 30 min prior to dark onset. The BioDAQ system was unavailable for this experiment. Instead, home cage food intake was assessed manually 1, 2, and 4h after pre-weighed chow was returned to the hopper at dark onset.

At least 21 days later, stress-induced hypophagia was assessed in a subset of the same mice (N = 8) using a mixed-model design with each mouse exposed to restraint stress only once. Chow access was removed 3 h prior to dark onset and all mice were injected with CNO (2 mg/kg, 5 ml/kg) 1 h before dark onset. Beginning 30 min before dark onset, mice were restrained in decapicones (MDC-200, Braintree Scientific) or left undisturbed for 30 min. Pre-weighed chow was returned to the hopper at dark onset when restrained mice were released from restraint, and cumulative food intake was manually measured 2h later.

#### 2.4.3 Terminal procedure

At least 12 days after assessment of stress-induced hypophagia, the same mice were injected with CNO (2 mg/kg, 5 ml/kg) 1.5–3 h into the light phase. Thirty minutes later they were exposed to one of two psychogenic stressors: 30 min restraint stress as above (n = 5M), or 20 min in a novel, brightly illuminated open field (50 × 50 cm, N = 8M). Mice were then returned to their home cage. Ninety minutes after stressor onset, mice were anesthetized and transcardially perfused with fixative as described in section 2.7.1.

### 2.5 Exp 4: retrograde tracing of brain-wide CRH inputs to the NTS

A retrogradely transported AAV encoding tdTomato in a Cre-dependent manner (AAVrg-CAG-FLEX-tdTomato, Table 2) was microinjected into the cNTS of CRH-ires-Cre mice (JAX stock #012704; N = 6F,4M) as described above (section 2.2.1). Three to five weeks after viral injection and 7 h after light onset, mice were restrained in a decapicone for 30 min (N = 1F, 2M) or left undisturbed in their home cages (1F, 2M). Ninety minutes after the onset of restraint stress, mice were anaesthetized and transcardially perfused as described below (section 2.7.1). Brains were extracted and processed for cFOS using immunofluorescence as described in section 2.7.2 and Table 3.

**Table 3:** Primary antibody details.

Target	Source and catalogue #	Antibody accession #	Dilution	Secondary antibody
cFOS	Cell signaling Technology, 9F6	AB_2247211	1:10,000 (IHC), 1:1000 (IF)	Biotin-conjugated anti-rabbit (IHC), AlexaFluor-647 (IF)
mCherry	Takara Bio, #632543	AB_2307319	1:2,000	AlexaFluor-488 anti-mouse, AlexaFluor-647 anti-mouse
dsRed	Takara Bio, #632496	AB_10013483	1:20,000 (IHC), 1:2,000 (IF)	Biotin-conjugated anti-rabbit (IHC), Cy3-conjugated anti-rabbit (IF), AlexaFluor-488 anti-rabbit
RFP	Synaptic systems #390 004	AB_2737052	1:2,000	555-Conjugated anti-Guinea pig
GFP, venus	Abcam, Ab13790	AB_300798	1:5,000	AlexaFluor-488 anti-chicken
GLP1	Bachem, T-4363	AB_518978	1:10,000	Biotin-conjugated anti-rabbit
Tyrosine hydroxylase	Millipore, AB152	AB_390204	1:2,000	Cy3-conjugated anti-rabbit
Oxytocin	PS36, gift from Harold Gainer [82]	–	1:500	Cy3-conjugated anti-mouse

### 2.6 Experiment 5: exogenous CRH into the lateral ventricle

#### 2.6.1 Cannula implantation

mGlu-Venus mice (N = 6F,6M) were anaesthetized using isoflurane (1–3%, 1.5 ml/min in O_2_) and placed in a stereotaxic frame. A 2 mm, 26G unilateral guide cannula (Plastics One) was implanted to target the lateral ventricle at the following coordinates from bregma: 250 μm caudal, 1 mm lateral, and 2.5 mm ventral. Following surgery, mice were allowed to recover for at least 16 days before undergoing behavioural testing.

#### 2.6.2 Open field and terminal procedure

Using a between-subjects design, mice were infused with CRH (1 μg in 1 μl saline, Tocris, #1151) or saline (1 μl) into the lateral ventricle. Fifteen minutes later, their exploratory behaviour was recorded in a novel open field as described in section 2.2.4. At least 24 h later, mice were infused again with CRH (N = 3F,3M) or saline (N = 3F,3M) and transcardially perfused 90 min later as described in section 2.7.1. Tissue was processed for Venus, Tyrosine hydroxylase (TH), and cFOS using immunofluorescence labelling as described in section 2.7.2 and Table 3.

### 2.7 Tissue collection and processing

#### 2.7.1 Transcardial perfusion

Mice were anaesthetized using pentobarbital sodium (US: Fatal Plus, 100 mg/kg, i.p.; Henry Schein; UK: Dolethal, 140 mg/kg, i.p.; Vetoquinol). They were then transcardially perfused with ice-cold phosphate buffer (PB, 0.1M, pH 7.2) followed by 4% paraformaldehyde in PB before brains were extracted for further processing (see sections 2.7.2–2.7.4).

#### 2.7.2 Immunohistochemical (IHC) and immunofluorescence (IF) labelling

After perfusion fixation, brains were immediately extracted and post-fixed overnight in 4% paraformaldehyde at 4 °C. Following cryoprotection in sucrose (20% in 0.1 M PB), coronal sections (30–35 μm) were collected on a freezing microtome and stored in cryopreservant solution [46] at −20 °C until further processing.

Sections were removed from cryopreservant and rinsed in four changes of 0.1M PB, followed by treatment with 0.5% sodium borohydride in PB for 20 min at room temperature. Sections destined for IHC (i.e., immunoperoxidase) labelling were further incubated in 0.15% hydrogen peroxide for 15 min at room temperature to suppress endogenous peroxidase activity. Pre-treated sections were then incubated overnight at room temperature (16–24h) in one of several primary antibodies (see Table 3) diluted in 0.1M PB containing 0.3% Triton-X and 1% normal donkey serum. For anti-GLP-1 labelling, sections were instead incubated for 1 h at room temperature followed by 60–65h at 4 °C. Sections were then rinsed in four changes of 0.1M PB over 1 h followed by incubation in species-specific secondary antibody (Table 3 and 1:500) at room temperature for 1 h (for IHC) or 2 h (for IF). After three rinses in 0.1M PB sections were either mounted onto glass slides (for IF) or incubated in Vectastain Elite avidin-biotin complex kit reagents diluted in 0.1M PB containing 0.3% Triton for 1.5–2h (for IHC). Peroxidase activity was localized using diaminobenzidine catalysed with hydrogen peroxide. Sections were then rinsed, mounted and left to dry. Following dehydration in increasing concentrations of ethanol, sections were cleared in xylene and coverslipped using Cytoseal 60 (Electron Microscopy Sciences, 18007).

#### 2.7.3 RNAscope *in situ* hybridization

Tissue sections were processed using fluorescence *in situ* hybridisation (FISH, RNAscope Multiplex Fluorescent Reagent Kit version 2, Advanced Cell Diagnostics [ACD], #323100) to label preproglucagon (*Ppg*), *Crhr1* and *Crhr2* mRNA transcripts in the cNTS and *Crh* mRNA transcripts in the PVN. Coronal sections containing the PVN or cNTS were pre-treated with hydrogen peroxide for 30 min (ACD, #323100) at room temperature, slide-mounted in dH_2_O (Fisherbrand SuperFrost Plus #12-550-15) and left to dry overnight at room temperature. Following 10 s dehydration in 100% ethanol, a hydrophobic barrier was drawn, and sections were treated with protease IV (ACD, #322336) for 25 min at room temperature. Following three rinses in dH_2_O, sections were left to incubate in RNAscope probe Mm-*Gcg* (ACDbio #482311, NM_008100.4; detects the mRNA *Ppg* encoded in the *Gcg* gene), Mm-*Crh* (ACDbio # 316091, NM_205769.2), Mm-*Crhr1* (ACDbio #418011, NM_007762.4) and Mm-*Crhr2* (ACDbio #413201, NM_009953.3) for 2h at 40 °C in the HybEZTM oven (ACD). Sections then underwent three amplification steps and labelling with Cy3-or Cy5-conjugated Tyramine Signal Amplification Plus (PerkinElmer; for *Crh, Gcg,* and *Crhr1*) or TSA-Vivid 650 (Bio-Techne #7527/1; for *Crhr2* and *Crhr1*) according to the ACD protocol. Slides were washed in wash buffer (ACD, #310091) 3 × 3 min between incubation steps. The same sections were subsequently processed as detailed above for immunofluorescent enhancement of GFP or tdRFP/mCherry reporter proteins.

#### 2.7.4 Light microscopy and cell counting

Images of single or dual IHC labelling were captured using a KEYENCE microscope (BZ-X700) and integrated software to generate focused images though the section thickness. IF and FISH labeling were visualized either on a KEYENCE microscope, a Zeiss widefield Axio Imager.M2 with a Solid-State Light Source (Zeiss Colibri 7; excitation wavelengths: 630, 590, 555, 475, and 385 nm) and a quadruple bandpass filter (emission filters: 425/30 + 514/30 + 592/25 + 709/100 nm), or on a Leica TCS SP8 confocal microscope using a 20× air objective and a 40× oil-immersion objective. AlexaFluor-488, Cy3, and AlexaFluor-647 were excited using a 488 nM OPSL, 552 nM OPSL, and 638 nm Diode laser, respectively. Confocal images were acquired sequentially using Leica LAS 4.0 image collection software. Brightness and contrast were adjusted using Fiji open source biological image analysis software [47]. All cell counts were performed manually in captured images. In Exp 3 and 5, counts of cFOS-IR nuclei in the cNTS (approximately 7.5–8.0 mm caudal to bregma) were made bilaterally using an average of 5 sections per mouse, with sections spaced by 105 μm.

### 2.8 Statistics

Statistical significance was assessed using null-hypothesis testing, including Student's T test and 3- and 2-way ANOVA, as indicated in the text and figures. Statistically significant interactions (p < 0.05) were followed up with Sidak's multiple comparisons test. Exact p-values for each test are indicated in graphs and/or figure legends. In each graph, individual data points are indicated either by a symbol (if unpaired) or by a line (if paired). In some graphs, males are indicated by triangle symbols and females by circles. To facilitate interpretation of the magnitude and precision of the results, we also include plots of relevant effect sizes (adjacent to each traditional bar graph) derived from estimation statistics, in which the mean difference between groups is plotted on a floating axis as a bootstrap sampling distribution. The mean difference is depicted as a cross (x); the 95% confidence interval is indicated by the vertical error bar. Effect size plots were generated using estimationstats.com [48].

## 3 Results

### 3.1 Exp 1: chemogenetic activation of PPG neurons elicits hypophagia and sex-dependent anxiety-like behaviour

#### 3.1.1 Selective chemogenetic activation of PPG neurons potently suppresses feeding by advancing meal termination

**Figure 1:**
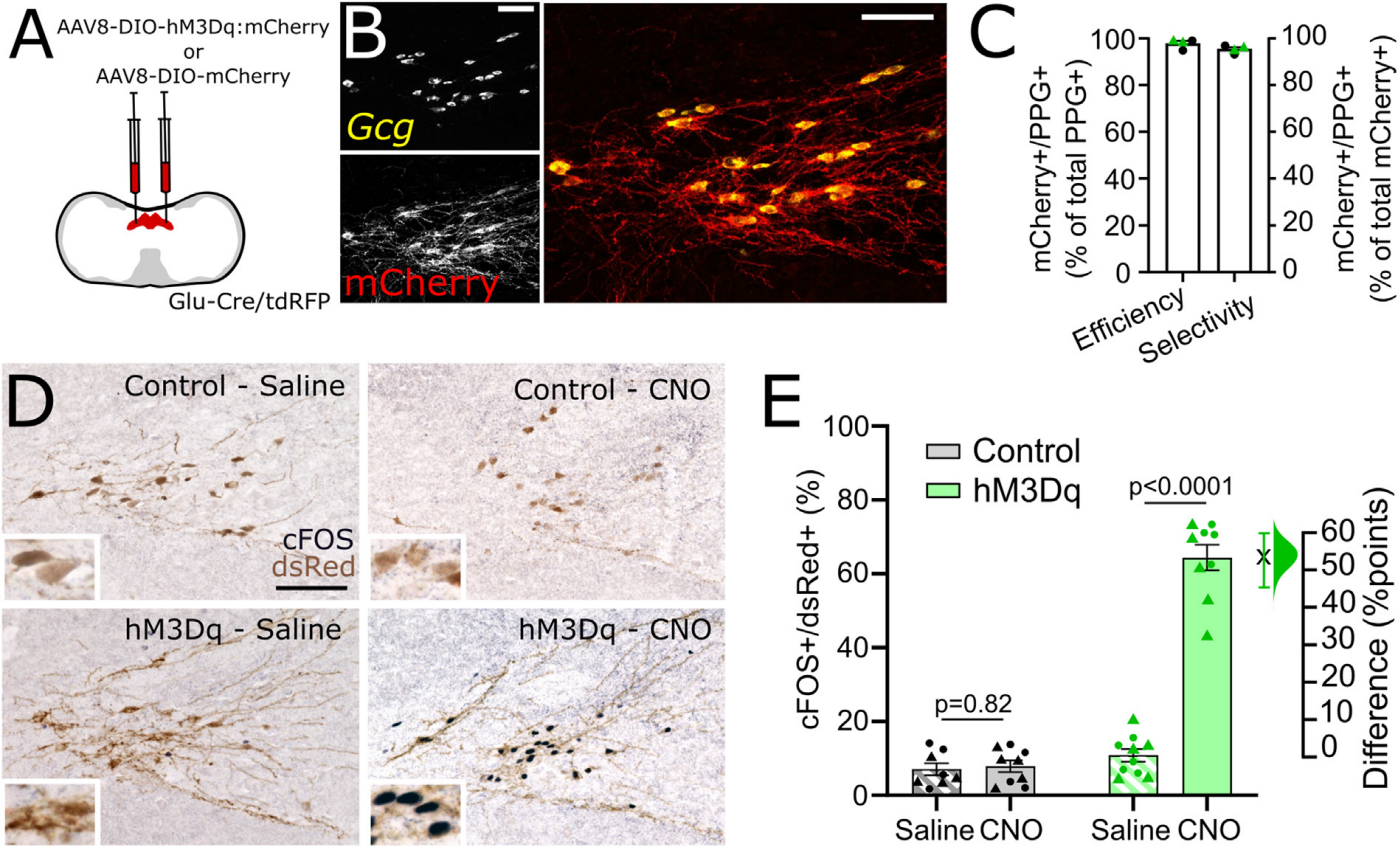
**Selective and efficient chemogenetic activation of cNTS PPG neurons *in vivo.*** A) Schematic of injection protocol. B) Representative images of RNAscope *in situ* hybridisation for *Ppg* mRNA (yellow) and immunolabelling for mCherry (to detect hM3Dq:mCherry, red) in tissue from one Glu-Cre/tdRFP mice injected with AAV8-DIO-hM3Dq:mCherry. Scale bars: 100 μm. C) Percent of *Ppg-*expressing neurons in the cNTS also expressing hM3Dq:mCherry (efficiency), as well as percent of mCherry-expressing cNTS neurons also expressing *Ppg* (selectivity). Results from control mice (expressing mCherry only, n = 2) are indicated with black circles, while results from hM3Dq-expressing mice (n = 2) are indicated with green triangles. D) Representative images of immunohistochemical labelling for cFOS (black nuclear label) and dsRed (brown cytoplasmic label, detecting mCherry and tdRFP) in mice expressing mCherry only (control, top panels) or hM3Dq:mCherry (hM3Dq, bottom panels) injected with saline (2 ml/kg, left panels) or CNO (2 mg/kg, 2 ml/kg; right panels). Scale bar: 100 μm. E) Percent of mCherry-expressing cNTS neurons also labelled for cFOS in control (grey/black) and hM3Dq-expressing (green) mice injected with saline (2 ml/kg, pattern) or CNO (2 mg/kg, filled). Data from females are indicated by circles; males are indicated by triangles. Also shown is Gardner-Altman estimation plot showing the mean difference in activated neurons between saline and CNO-injected hM3Dq-expressing mice. Two-way drug × virus interaction: F(1,32) = 138.9, p < 0.0001. (For interpretation of the references to color in this figure legend, the reader is referred to the Web version of this article.)

**Figure S1:**
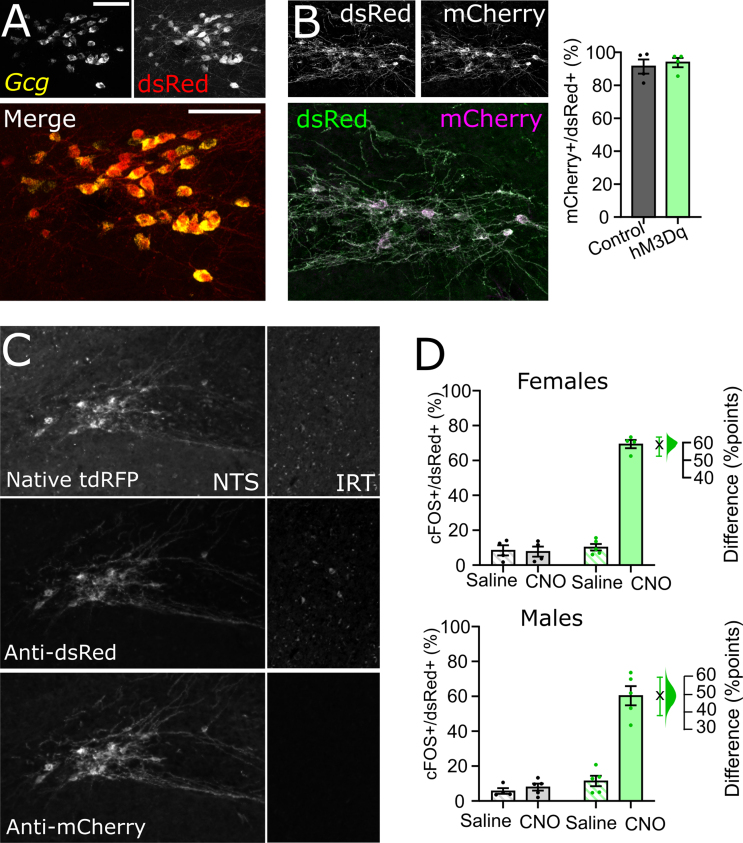
**Relating to**Figure 1. A) Immunolabelling for tdRFP (dsRed, red) and RNAscope *in situ* hybridisation for *Ppg* mRNA (yellow) in the cNTS of naïve Glu-Cre/tdRFP transgenic mice showing robust *Ppg* expression in tdRFP-positive cells with no discernible expression in non-*Ppg* cells in the cNTS. Scale bars: 100 μm. B) immunolabelling for mCherry (detecting hM4Di:mCherry only, magenta) and dsRed (detecting both hM4Di:mCherry *and* tdRFP, green) in Glu-Cre/tdRFP mice injected with AAV8-hSyn-DIO-hM3Dq:mCherry. Student's T-test: p = 0.66. C) Validation of selectivity of the mCherry antibody. Glu-Cre/tdRFP transgenic mice were injected into the cNTS, but not the intermediate reticular nucleus (IRT), which contains another population of PPG neurons, with AAV8-hSyn-DIO-hM3Dq:mCherry. Anti-dsRed labelling (detecting both mCherry and tdRFP, AlexaFluor-488 secondary) was seen in both the cNTS and IRT, while anti-mCherry (detecting mCherry only, AlexaFluor-647) labelling was only seen in the cNTS, where hM3Dq:mCherry was expressed. D) Data from Figure 1E grouped by sex. Percent of mCherry-expressing cNTS neurons also labelled for cFOS in male and female control mice (grey/black) and hM3Dq-expressing mice (green) injected with saline (2 ml/kg, striped) or CNO (2 mg/kg, solid) expressing mCherry only or hM3Dq:mCherry (hM3Dq) after being injected with saline (2 ml/kg) or CNO (2 mg/kg, 2 ml/kg). Gardner-Altman estimation plots display the estimated effect sizes. There was no three-way interaction of virus x drug x sex [F(1, 28) = 2.151; p = 0.1537].

We first verified the selectivity of the transgenic mGlu-Cre/tdRFP mouse model using FISH to detect *Ppg* mRNA (encoded in the *Gcg* gene). Cre-conditional fluorescent reporter tdRFP was fully colocalised with *Ppg* mRNA expression within the cNTS (Fig. S1A). Injection of AAV8-DIO-hM3Dq:mCherry or AAV8-DIO-mCherry into the cNTS (Figure 1A) led to high transduction efficiency and selectivity (Figure 1B,C) indicated by colocalization of mCherry (viral transgene detected with an mCherry-selective antibody, Fig. S1C) and *Ppg* mRNA (Figure 1B,C), with no difference in transduction efficiency between the two viruses (Fig. S1B). Compared to saline injection, CNO (2 mg/kg, i.p.) increased the percentage of cFOS-positive PPG neurons in the cNTS of hM3Dq-expressing mice by 52 percentage points (95% confidence interval [CI]: 41.9 to 61.5 percentage points; Figure 1D,E). This CNO effect was independent of sex (Fig. S1D) and was absent in virus control mice (95%CI: -4.05 to 4.95 percentage points; Figure 1D,E).

**Figure 2:**
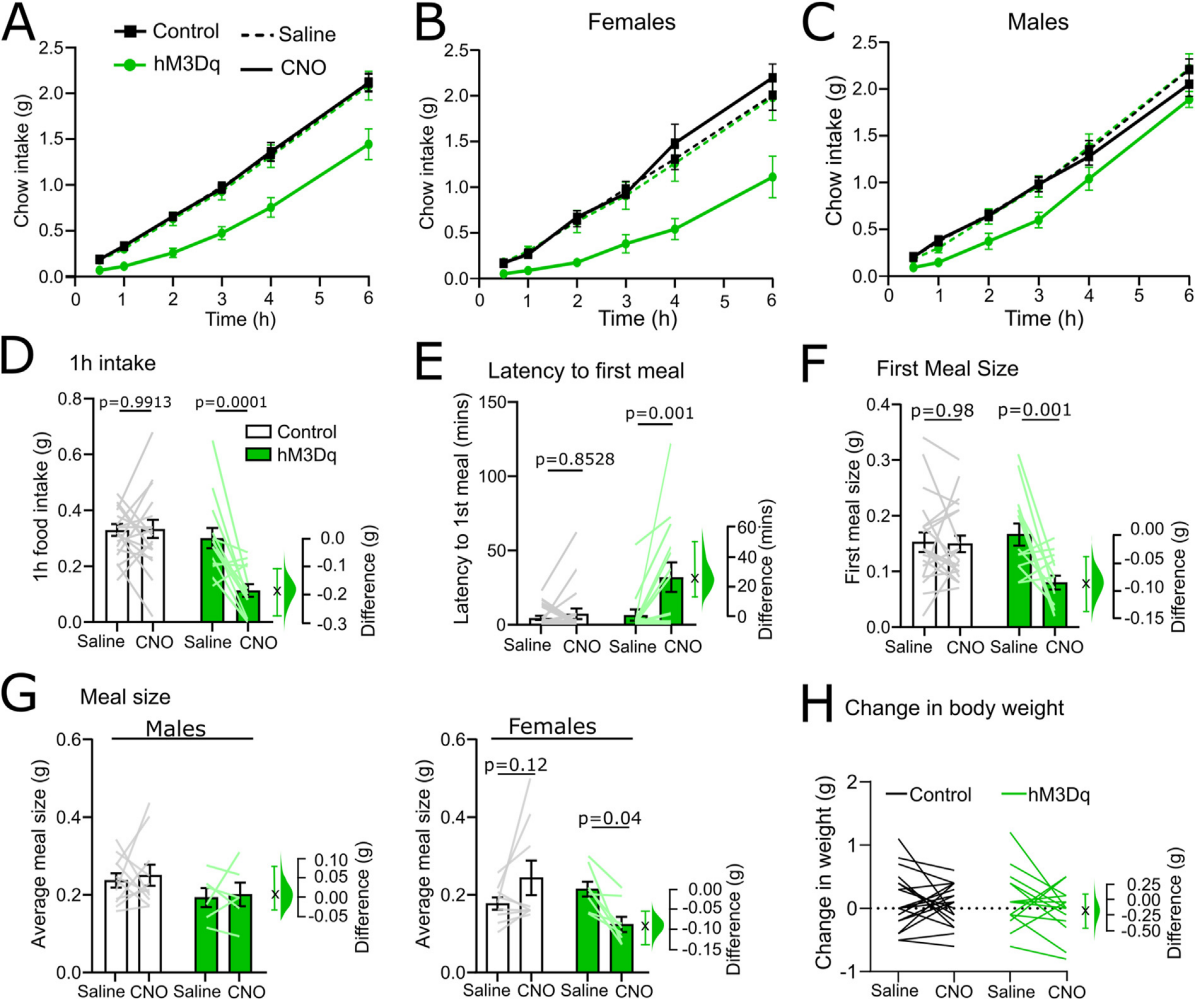
**Meal pattern analysis of chow intake following chemogenetic activation of cNTS PPG neurons *in vivo.*** A) Cumulative chow intake over the first 6 h of the dark phase of male and female control (black squares) and hM3Dq-expressing mGlu-Cre/tdRFP mice (green circles) following i.p. injection of either saline (dashed lines, 2 ml/kg) and CNO (solid lines, 2 mg/kg, 2 ml/kg). Three-way drug x time × virus interaction: F(5,110) = 3.628, p = 0.0045. B) Cumulative chow intake of females only. Three-way drug x time × virus interaction: F(5,70) = 6.889, p < 0.0001. Two-way drug x time (hM3Dq): F(5,35) = 9.625, p < 0.0001. Two-way drug x time (control): F(1.499, 10.49) = 0.8945, p = 0.4088. C) Cumulative chow intake of males only. Three-way drug x time × virus interaction: F(5,75) = 0.7909, p = 0.5595. Main effect of time [F(1.429, 21.43) = 436.3, p < 0.0001]. D) Chow intake by male and female mice 1 h after dark onset [drug x virus: F(1, 32) = 13.17; p = 0.0010]. E) Latency of male and female mice to begin feeding [drug x virus: F(1, 32) = 7.058, p = 0.0122]. F) Size of the first meal in male and female mice [drug x virus: F(1, 32) = 7.895; p = 0.0084]. G) Average meal size over the first 6 h grouped by sex in control (white bars) and hM3Dq-expressing mice (green bars) injected with saline (2 ml/kg) or CNO (2 mg/kg, 2 ml/kg). Three-way drug x virus × sex interaction: F(1, 30) = 4.882; p = 0.0349; two-way drug x virus (females): F(1, 15) = 11.11, p = 0.0045; two-way drug x virus (males): F(1, 15) = 0.01089, p = 0.9183). H) No significant change in bodyweight of control (black) or hM3Dq-expressing mice (green) 24 h after injection of saline (2 ml/kg) or CNO (2 mg/kg, 2 ml/kg) [virus x drug: F(1, 32) = 0.4636, p = 0.5008]. The difference in the relevant outcome between saline and CNO-injected hM3Dq-expressing mice is shown in D-H using Gardner-Altman estimation plots. (For interpretation of the references to color in this figure legend, the reader is referred to the Web version of this article.)

**Figure S2:**
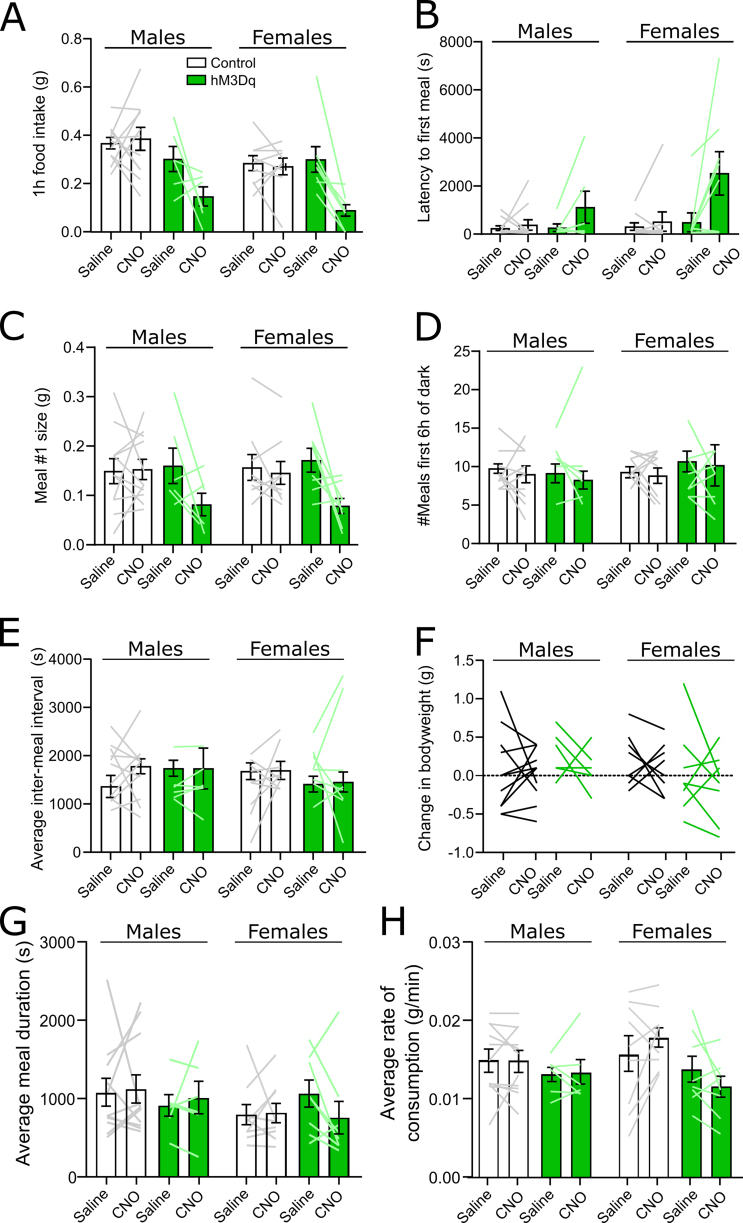
**Relating to**Figure 2. Meal pattern data grouped by sex in control mice (black) and hM3Dq-expressing mice (green) following injection of saline (2 ml/kg) or CNO (2 mg/kg, 2 ml/kg). A) One hour food intake; no three-way drug x sex × virus interaction [F(1, 30) = 0.05197; p = 0.8212]. B) Latency to first meal; no three-way drug x sex × virus interaction [F(1, 30) = 0.05197; p = 0.8212]. C) First meal size; no three-way drug x sex × virus interaction [F(1, 30) = 8.890e-005; p = 0.9925]. D) Number of meals over the first 6 h of the dark phase; no three-way drug x sex × virus interaction: F(1,30) = 0.009932, p = 0.9213. E) Average inter-meal interval over the first 6 h of the dark phase; no three-way drug x sex × virus interaction: F(1,30) = 0.009742, p = 0.9220; no main effects F) Change in body weight 24 h after CNO or saline injection; no three-way drug x sex × virus interaction [F(1,30) = 0.008697, p = 0.9263] and no main effects. G) Average meal duration over the first 6 h of the dark phase; no three-way drug x sex × virus interaction: F(1,30) = 0.9161, p = 0.3461; no main effects. H) Average rate of consumption over the first 6 h of the dark phase; no three-way drug x sex × virus interaction: F(1,30) = 2.811, p = 0.1040; no main effects.

When data from male and female mice were combined, chemogenetic activation of PPG neurons suppressed cumulative food intake during 6 h after dark onset (Figure 2A), consistent with previous findings [21,[23], [24], [25]]. This prolonged suppression in feeding was driven primarily by females eating significantly less following chemogenetic activation of PPG neurons (Figure 2B; treatment x virus × time interaction: F(5, 70) = 6.889, p < 0.0001). In contrast, there was no significant three-way interaction in male mice [Figure 2C,F(5, 75) = 0.7909, p = 0.56]. However, at 1 h after dark onset, feeding was suppressed to a similar degree in both sexes following chemogenetic activation of cNTS^PPG^ neurons (Fig. S2A, Figure 2D, effect size: -0.187g [95%CI: -0.278g, −0.109g]). The latency to initiate the first meal was increased by 25.5 min ([95%CI: 12.3 min, 49.3 min], Fig 2E) following chemogenetic activation, with no effect of sex (Fig. S2B). Furthermore, the size of that first meal was reduced by 0.086g ([95%CI: 0.039g, 0.137g], Fig 2F), with no effect of sex (Fig. S2C). There was no effect of CNO on the number of meals over the first 6 h (Fig. S2D), the average time taken between meals (Fig. S2E), the average duration of meals over the first 6 h (Fig. S2G), or the rate of consumption (Fig. S2H). Interestingly, cNTS^PPG^ neuron activation reduced average meal size by 0.091g (95%CI: 0.138g, 0.054g) in female mice over the first 6h of the dark phase, whereas the effect in males was more transient (Figure 2C,G). Chemogenetic activation had no impact on bodyweight 24 h after injection of CNO (Figure 2H, Fig. S2F), and CNO did not impact bodyweight or any assessed food intake metric in virus control mice.

#### 3.1.2 Activation of PPG neurons is sufficient to induce mild anxiety-like behaviour

**Figure 3:**
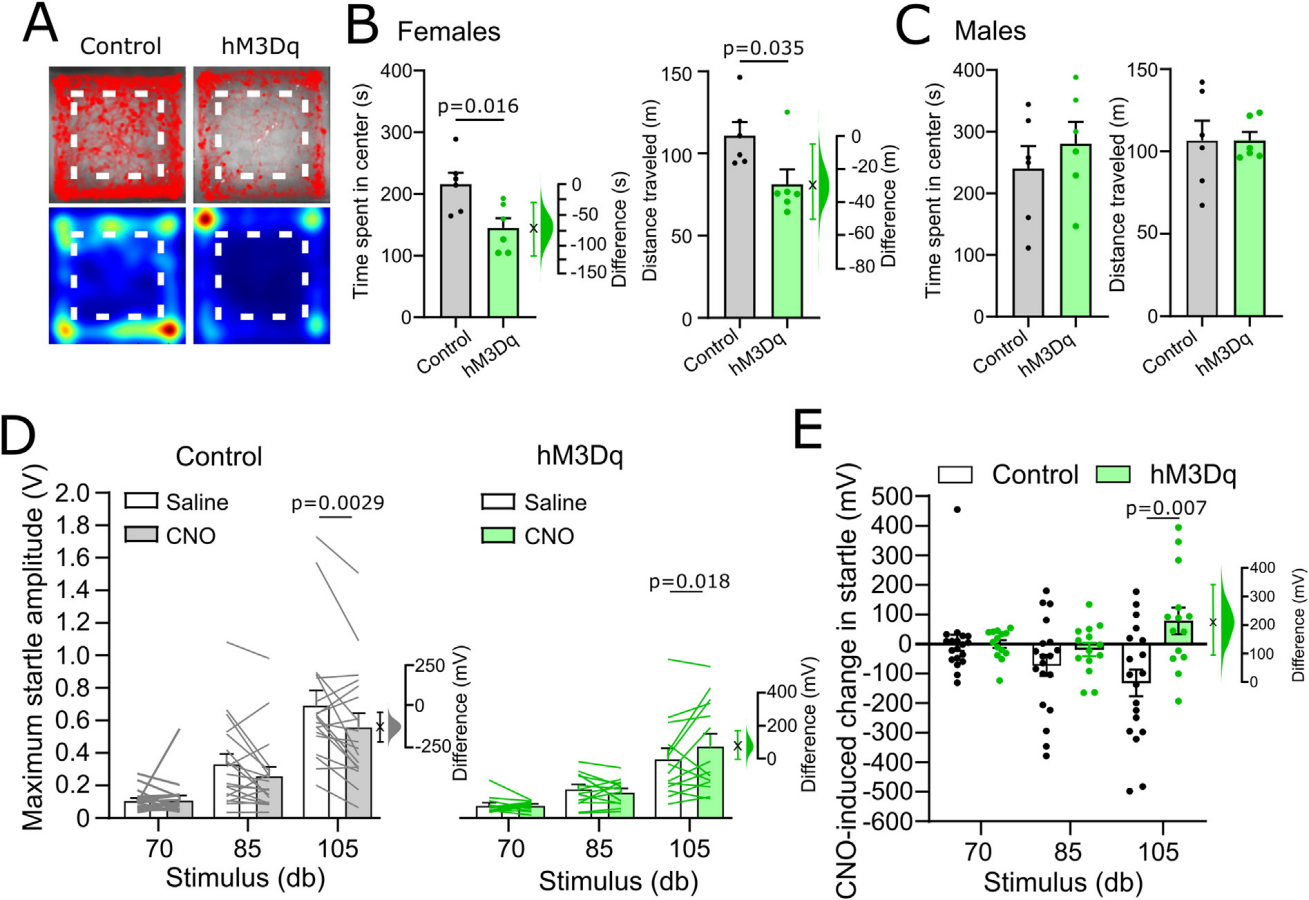
**Chemogenetic activation of cNTS PPG neurons is sufficient to elicit moderate increases in anxiety-like behaviour.** A) Traces and heatmaps from a representative control (left) and hM3Dq-expressing mouse (right) recorded in the open field following injection of CNO (2 mg/kg, 2 ml/kg). The centre of the arena is outlined using a dashed line. B–C) Quantification of time spent in the centre of the open field (left) and the total distance travelled (right) in female (B) and male (C) control (white bars) and hM3Dq-expressing mice (green bars). Student's T-test: females, centre time: t = 2.912, df = 10; females, distance: t = 2.438, df = 10; males, centre time: t = 0.7911, df = 10; males, distance: t = 0.003524, df = 10. D) Maximum acoustic startle amplitude of control (grey/white) and hM3Dq-expressing (green) male and female mice (combined due to no sex difference [Fig. S3]) following injection of saline (2 ml/kg) or CNO (2 mg/kg, 2 ml/kg). Three-way drug x virus × db interaction: F(2, 62) = 5.233; p = 0.0079. Two-way drug × db interaction (control): F(2, 36) = 3.435; p = 0.0431. Two-way drug × db interaction (hM3Dq): F(2, 26) = 3.904; p = 0.0329. E) CNO-induced startle calculated as the difference in startle between injection of saline and CNO for each animal. Control mice: black circles, white bars; hM3Dq-expressing mice: green circles, green bars. Two-way virus × db interaction: F(2, 62) = 5.233; p = 0.0079. Also shown in B), D) and E) is the effect size using a Gardner-Altman estimation plot. (For interpretation of the references to color in this figure legend, the reader is referred to the Web version of this article.)

Increased latency to begin feeding and short-term suppression in food intake can be indicative of negative affect, including anxiety-like states [1]. While a previous study failed to find any impact of chemogenetic activation of cNTS^PPG^ neurons on anxiety-like behaviours in male mice [23], results from several studies in rats have revealed anxiogenic effects of GLP-1 receptor stimulation in multiple brain regions [33,36,37]. Given this apparent discrepancy in the rodent literature, we next investigated the extent to which chemogenetic activation of cNTS^PPG^ neurons is sufficient to induce anxiety-like behaviour in male and female mice using two behavioural assays. First, we analysed exploratory behaviour of male and female mice placed into a novel open field, a validated test for anxiety-like behaviour that relies on rodents’ innate avoidance of open, exposed areas and their tendency to stay close to corners and edges [49,50]. We found that female and male mice behaved differently in the open field (main effect of sex: p = 0.03), with males exhibiting higher variability in the time spent in the centre of the field (Figure 3C). In female mice, chemogenetic activation of cNTS^PPG^ neurons significantly reduced the time spent in the centre of the open field by 71s (95%CI: 29.3s, 116s; Figure 3A,B), and reduced the total distance travelled by 29.5m (95%CI: 0.5m, 48.5m, Figure 3A,B). In male mice, chemogenetic activation had no effect on time spent in the centre of the open field or on total distance travelled (Fig 3C).

**Figure S3:**
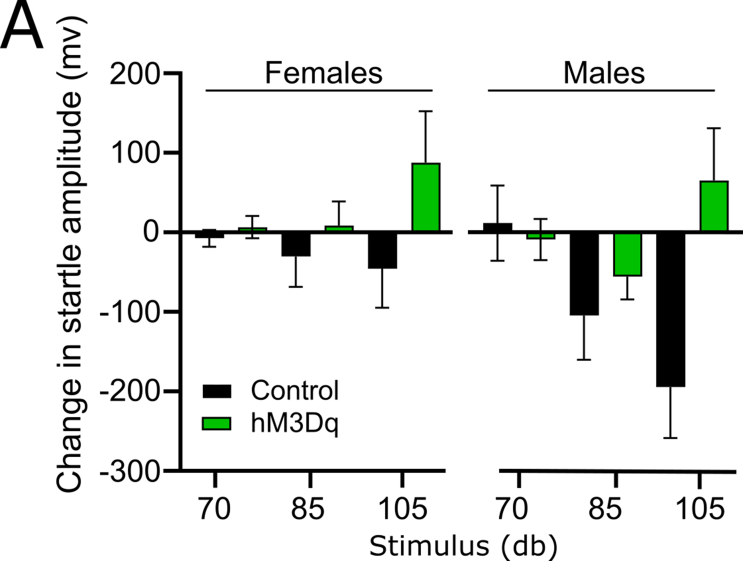
**Relating to**Figure 3. Acoustic startle results in female and male mice. A) Treatment effects on acoustic startle calculated as the within-subjects difference in startle amplitude in mice after injection of saline vs. CNO. No three-way drug x sex × virus interaction [F(2,58 = 0.721, p = 0.491].

A second test investigated anxiety-like behaviour in male and female mice by assaying acoustic startle response magnitude, which does not depend on locomotion or exploratory behaviour. Another benefit of the acoustic startle test is that it can be conducted using a repeated-measures, within-subject design [51]. There was no significant difference between males and females in the startle response to three different noise (db) levels (main effect of sex control group: p = 0.21, main effect of sex hM3Dq group: p = 0.61). However, there was a significant three-way drug x virus × db interaction [F(2, 62) = 5.233, p = 0.0079, Figure 3D]. Follow-up analyses revealed a significant two-way drug × db interaction in both the control [F(2, 36) = 3.435, p = 0.043)] and hM3Dq group [F(2, 26) = 3.904, p = 0.033]. CNO (2 mg/kg i.p.) reduced startle responses at the highest noise level (105 db) in control mice (effect size: -132 mV with a 95%CI of [−40.04 mV, −223.6 mV]), consistent with a previous report in Long-Evans rats treated with CNO [52]. This effect of CNO on acoustic startle appeared to be more pronounced in male mice, but the effect of sex was not significant (Fig. S3). Conversely, in mice expressing hM3Dq, CNO injection (2 mg/kg) *increased* startle amplitude responses to the 105 db noise by 78.1 mV (95%CI: -2.31 mV, 168 mV). This differential CNO effect in control and hM3Dq mice became even more apparent when the difference between startle amplitude in the presence of saline vs. CNO was plotted: CNO-induced increases in startle amplitude within subjects was 210 mV higher in hM3Dq mice compared to control mice (95%CI: 96.8 mV, 337 mV, Fig 3E).

### 3.2 Exp 2: rabies virus-mediated circuit tracing reveals stress-activated cNTS^PPG^-projecting PVN neurons

**Figure 4:**
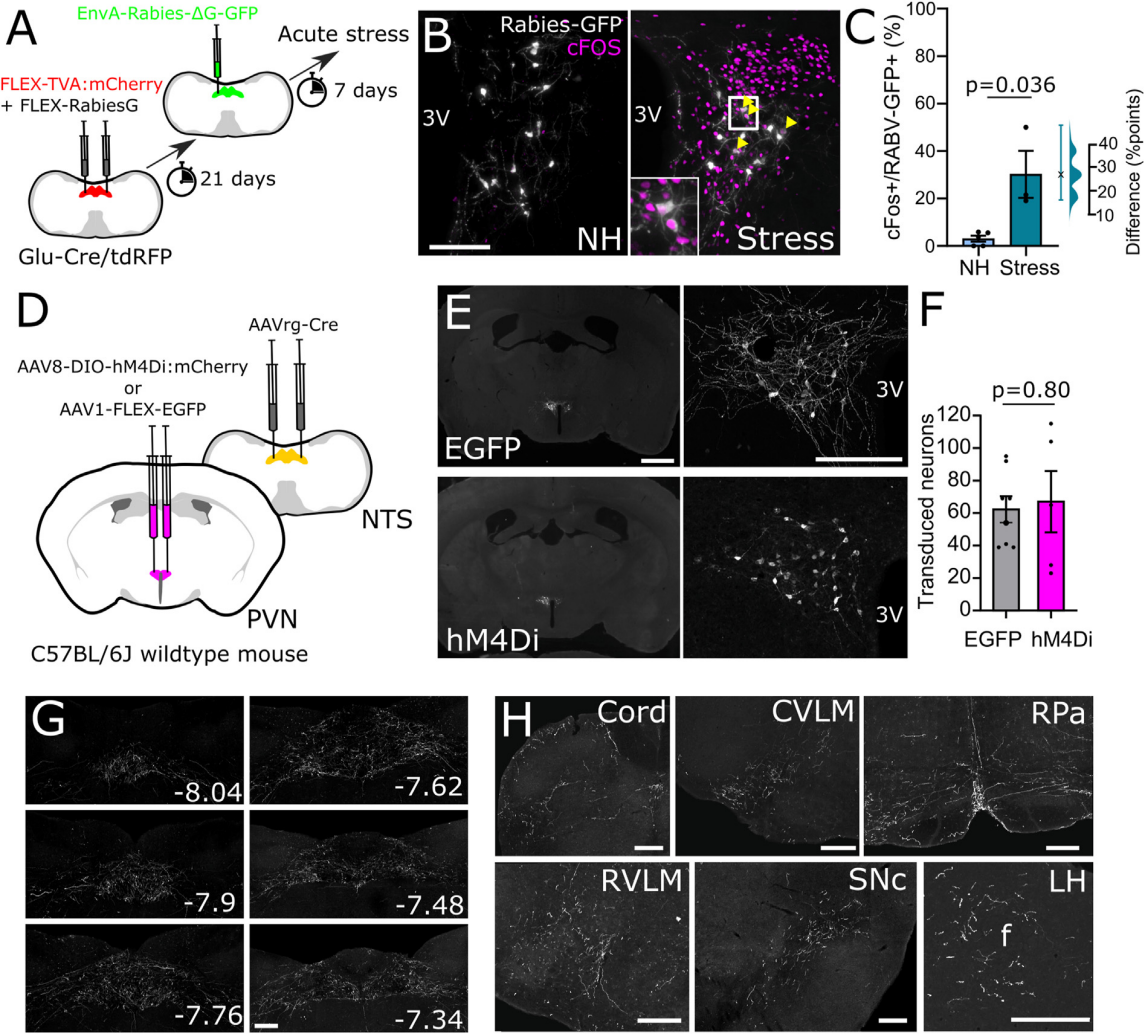
**Identification and transduction of a stress-activated PVN→NTS circuit targeting PPG neurons.** A) Diagram showing strategy to label stress-activated monosynaptic input from the PVN to cNTS PPG neurons. B) GFP immunofluorescence (greyscale) in the PVN 7 days after unilateral microinjection of (EnvA)-RABV-ΔG-GFP targeted to the cNTS. cFOS immunoreactivity (magenta nuclei, pseudocolour) and RABV-GFP immunofluorescence (greyscale) is shown in a representative non-handled mouse (NH, left) and in a mouse perfused 90 min after the onset of 30 min restraint stress (Stress, right). Scale bar: 100 μm. C) Calculated percentage of RABV-GFP labelled cells that were also cFOS-positive in nonhandled mice (-, n = 5) vs mice exposed to 30 min restraint stress (n = 3). Mann–Whitney U test: t = 3.203, df = 5. Also shown is the difference between EGFP and hM4Di-expressing mice in the percentage of RABV-GFP labelled cells activated to express cFOS using a Gardner-Altman estimation plot. D) Schematic illustrating the intersectional approach for targeting of NTS-projecting PVN neurons to induce Cre-dependent expression of EGFP or hM4Di. E) Representative images at low (left, scale bar: 1 mm) and high magnification (right, scale bar: 200 μm) of immunolabelling for EGFP (top) or hM4Di:mCherry (bottom) in NTS-projecting PVN neurons. 3V: third ventricle. F) Similar numbers of NTS-projecting PVN neurons transduced to express either EGFP or hM4Di:mCherry. G) Representative images of PVN-derived, EGFP-labelled axons throughout the cNTS in mice expressing EGFP in NTS-projecting PVN neurons. Scale bar: 100 μm. Numbers indicate distance from bregma in mm. H) Additional axon collateral targets of NTS-projecting PVN neurons. Cord: spinal cord, CVLM: caudal ventrolateral medulla, RPa: raphe pallidus, RVLM: rostral ventrolateral medulla, SNc: substantia nigra pars compacta, LH: lateral hypothalamus. Scale bars: 100 μm.

**Figure S4:**
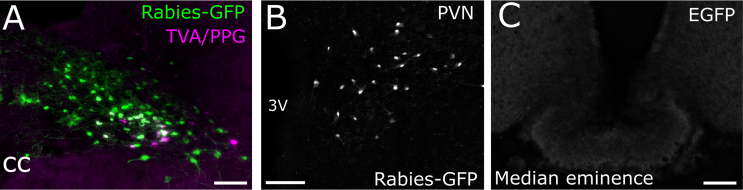
**Relating to**Figure 4. A) Representative image of RABV-GFP labelling (green) and dsRed-positive cells (magenta) expressing TVA receptor and/or tdRFP within the NTS. Scale bar: 100 μm. B) RABV-GFP labelling in the PVN following monosynaptic tracing from PPG neurons. Scale bar: 100 μm. C) No detectable axon collaterals within the median eminence arising from NTS-projecting PVN neurons. Scale bars: 100 μm.

Hypophagia and anxiety-like behaviours are driven by neural circuits whose activity is modulated by acute and chronic stress [3,6,36,53]. Having revealed a potential role for cNTS^PPG^ neurons not only in food intake but also in anxiety-like behaviour in mice, we next sought to identify neural pathways which may drive activation of cNTS^PPG^ neurons in response to acute stress. Rabies-mediated monosynaptic tracing (Figure 4A, Fig. S4A) confirmed our previous findings that multiple forebrain, midbrain, and hindbrain regions provide direct input to cNTS^PPG^ neurons [10], with particularly prominent afferent inputs arising from the PVN (Fig. S4B). Prior to perfusion, mice were exposed to 30 min restraint stress or left as non-handled controls (NH) Compared to NH controls, restraint stress increased the percentage of cNTS^PPG^-projecting PVN neurons expressing cFOS by 27.1 percentage points (95%CI: 16.1, 47.8 percentage points; Figure 4B,C), supporting a role for PVN neurons in stress-induced activation of postsynaptic cNTS^PPG^ neurons.

### 3.3 Exp 3: chemogenetic inhibition of NTS-projecting PVN neurons

#### 3.3.1 Monosynaptic input from the PVN drives cNTS^PPG^ neuron activation in male mice

We next used an intersectional approach to acutely inhibit NTS-projecting PVN neurons in male wildtype mice, including (but not limited to) neurons that provide synaptic input to cNTS^PPG^ neurons. For this, a retrogradely transported AAV (AAVrg-hSyn-Cre) was microinjected into the NTS in male C57BL/6J mice to induce Cre expression in NTS-projecting neurons throughout the brain, combined with injection of AAV8-hSyn-DIO-hM4Di:mCherry or AAV1-CAG-FLEX-EGFP into the PVN to induce selective expression of hM4Di:mCherry or EGFP (as control) in NTS-projecting PVN neurons (Figure 4D,E). There was no difference in the number or distribution of neurons transduced with either virus (Fig 4F). In virus control mice, EGFP labelling filled the cytoplasm and processes of transduced PVN neurons, including their axon collaterals, revealing other brain regions that receive collateralized input from NTS-projecting PVN neurons. As expected, the NTS contained the densest accumulation of axonal GFP-immunolabelling, which extended through the rostrocaudal extent of the cNTS (Fig 4G). Additional GFP-immunolabelled fibres representing the axon collaterals of NTS-projecting PVN neurons were seen in the spinal cord, the caudal ventrolateral medulla, the raphe pallidus, the rostral ventrolateral medulla, the substantia nigra, and the lateral hypothalamus (Fig 4H). No GFP-positive fibres were observed within telencephalon regions, within diencephalic regions rostral to the PVN, or within the median eminence (Fig. S4C).

**Figure 5:**
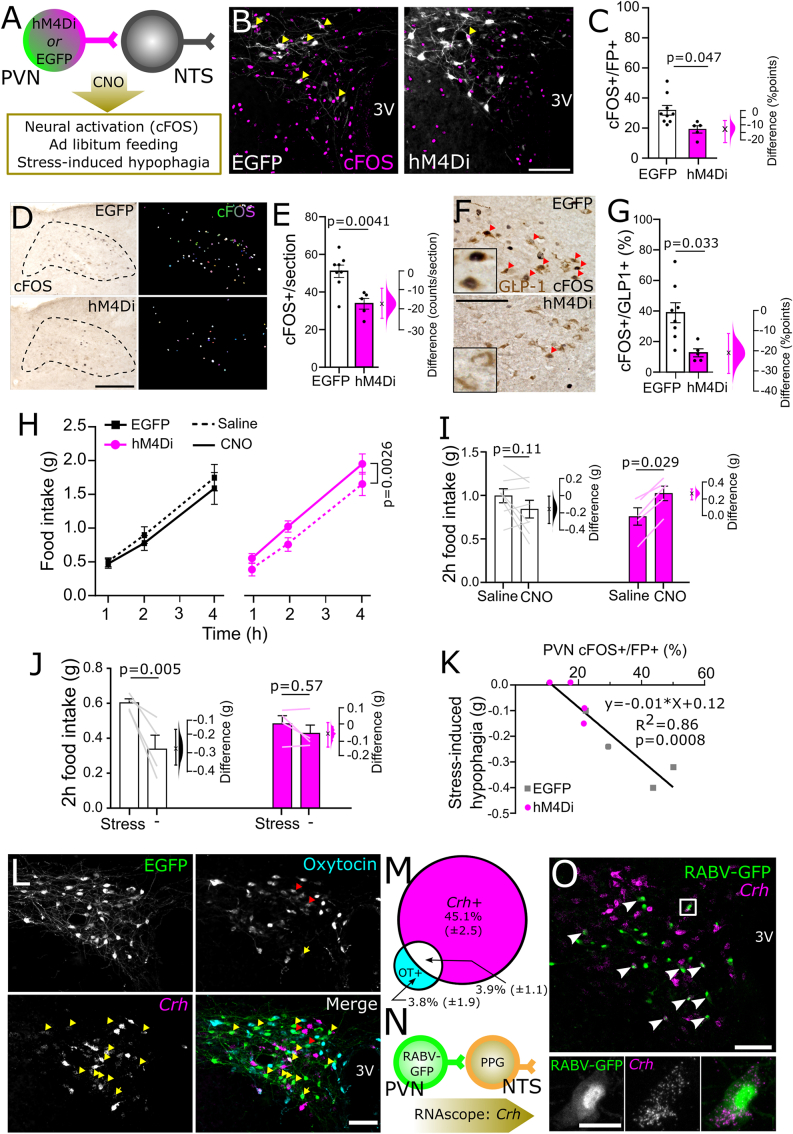
**Chemogenetic inhibition of NTS-projecting PVN neurons.** A) Diagram of the experimental paradigm for chemogenetic inhibition of NTS-projecting PVN neurons. B) cFOS immunoreactivity (magenta nuclei, pseudocolour) and EGFP or mCherry immunofluorescence (greyscale) labelling in the PVN of a representative control mouse (left, EGFP) and a representative hM4Di-expressing mouse (right, hM4Di) after i.p. injection of CNO (2 mg/kg, 2 ml/kg) followed by 30 min restraint stress. 3V: third ventricle. Scale bar: 100 μm. C) Calculated percentage of EGFP- vs. hM4Di:mCherry-expressing (fluorescent protein, FP+) cells that were also cFOS-positive in mice after i.p. injection of CNO (2 mg/kg, 2 ml/kg) followed by exposure to an acute stressor (novel environment or restraint stress). Unpaired T-test: t = 2.498, df = 6. D) Representative images of cFOS-immunoreactivity in the cNTS of mice expressing EGFP (top) or hM4Di (bottom) in NTS-projecting PVN neurons. Counted cFOS-immunoreactive cells indicated with rainbow-coloured mask to the right. All mice were injected with CNO (2 mg/kg, 2 ml/kg) and then exposed to novel environment or restraint stress. Scale bar: 100 μm. E) Counts of cFOS-immunoreactive nuclei in the cNTS in mice expressing EGFP or hM4Di in NTS-projecting PVN neurons and injected with CNO (2 mg/kg, 2 ml/kg) prior to stress exposure. Unpaired T-test: t = 3.603, df = 11. F) cFOS- and GLP-1-immunoreactivity in the cNTS of mice expressing EGFP (top) or hM4Di (bottom) in NTS-projecting PVN neurons and injected with CNO (2 mg/kg, 2 ml/kg) prior to stress exposure. Scale bar: 100 μm. G) Calculated percentage of GLP-1-immunoreactive cells in the cNTS that were also cFOS-IR. Unpaired T-test: t = 2.766, df = 6. H) Cumulative chow intake over 4 h after dark onset in mice expressing EGFP or hM4Di in NTS-projecting PVN neurons after injection with saline (2 ml/kg; dashed lines) or CNO (2 mg/kg, 2 ml/kg; solid lines). I) Chow intake over the first 2 h of dark phase in mice expressing EGFP or hM4Di in NTS-projecting PVN neurons after injection with saline (2 ml/kg) or CNO (2 mg/kg, 2 ml/kg). J) Chow intake during the first 2 h of dark onset of mice expressing EGFP or hM4Di in NTS-projecting PVN neurons and injected with CNO (2 mg/kg, 2 ml/kg) prior to 30 min restraint stress. Virus × stress interaction: F(1, 6) = 7.805, p = 0.0314. K) The relationship between activation of NTS-projecting PVN neurons and stress-induced hypophagia. The effect of stress on 2 h food intake in mice expressing EGFP (grey squares) or hM4Di (magenta circles) in NTS-projecting PVN neurons is plotted against the percentage of cFOS-positive NTS-projecting PVN neurons. L) Representative images of RNAscope *in situ* hybridisation for *Crh* (magenta in merged image) combined with immunolabelling for GFP (green in merged image) and oxytocin (cyan in merged image) in the PVN of mice expressing GFP in NTS-projecting PVN neurons. Scale bar: 100 μm. Yellow arrowheads indicate *Crh-*positive EGFP-labelled neurons; red arrows indicate oxytocin-positive EGFP-labelled neurons; yellow arrows indicate EGFP-labelled neurons positive for both *Crh* and oxytocin. M) Percentage of NTS-projecting PVN neurons expressing *Crh* and/or oxytocin (OT+). N = 3 mice, 2 sections containing the PVN from each mouse. N) Diagram illustrating RNAscope *in situ* labelling for *Crh* in cells with direct projections to NTS PPG neurons (RABV-GFP labelled). O) RNAscope *in situ* hybridisation for *Crh* in PPG-projecting PVN neurons (RABV-GFP labelled). Scale bar top panel: 100 μm. White arrowheads indicate *Crh*-positive RABV-GFP labelled neurons. Bottom panel: higher magnification image of inset in top panel. Scale bar: 20 μm. Gardner-Altman estimation plots in panels C, E, G, I, and J display estimated effect sizes for each assessed parameter. (For interpretation of the references to color in this figure legend, the reader is referred to the Web version of this article.)

To determine the extent to which recruitment of NTS-projecting PVN neurons contributes to stress-induced activation of cNTS^GLP−1/PPG^ neurons, EGFP and hM4Di-expressing mice were injected with CNO to inhibit NTS-projecting PVN neurons. Mice were then exposed to either 30 min restraint or 20 min in a novel environment, both of which are acutely stressful to rodents [54] (Figure 5A). There was no difference between stressors in their ability to activate cFOS in the cNTS (t = 1.088, df = 6, p = 0.32) or in NTS-projecting PVN neurons (t = 0.52, df = 6, p = 0.62), and so data were pooled for further analysis. As expected, CNO-induced chemogenetic inhibition attenuated stress-induced activation of hM4Di-expressing PVN neurons by 13.6 percentage points (95%CI: 7.37, 23.2 percentage points) compared to PVN activation in EGFP-expressing controls (Figure 5B,C). Further, acute inhibition of NTS-projecting PVN neurons was sufficient to reduce stress-induced activation of neurons within the cNTS by 19.4 counts/section (95%CI: 9.9, 29.4 counts/section, Figure 5D,E). The effect was even larger among GLP-1-positive cNTS neurons, for which the proportion of GLP-1 neurons expressing cFOS was reduced by 26.3 percentage points (95%CI: 13.7, 39.6 percentage points, Figure 5F,G). These results provide evidence that NTS-projecting PVN neurons contribute to stress-induced activation of PPG/GLP-1 and other cNTS neurons.

#### 3.3.2 NTS-projecting PVN neurons suppress feeding and drive stress-induced hypophagia

Chemogenetic inhibition of PVN neurons that project to the NTS was by itself sufficient to increase chow intake over the first 4 h of dark onset (Figure 5H, main effect of drug in hM4Di group: p = 0.0026). Two hours into the dark cycle, chow intake was increased by 0.266g [95%CI: 0.19, 0.316] in hM4Di-expressing male mice injected with CNO compared to saline (Fig 5I).

Since we previously found that NTS-projecting PVN neurons are activated in response to acute stress [10], we next tested whether chemogenetic inhibition of the PVN→NTS pathway was sufficient to reduce or block restraint stress-induced hypophagia in male mice. There was a large and statistically significant effect of restraint stress to reduce subsequent food intake in control mice, which consumed 0.265 g less over 2 h following the stressed vs. non-stressed condition (Figure 5J; 95%CI: 0.155, 0.36g). In contrast, restraint stress-induced hypophagia was significantly attenuated in mice with acute inhibition of NTS-projecting PVN neurons, with mice eating only marginally less during the 2-h period after restraint stress vs. the non-stressed condition (effect size: 0.055 [95%CI: -0.01, 0.135g]). The magnitude of stress-induced hypophagia was negatively correlated with the percentage activated NTS-projecting PVN neurons (R^2^ = 0.86, p = 0.0008), such that higher levels of activation of this pathway coincided with more pronounced food intake suppression (Fig 5K). These data support the hypothesis that a pathway from the PVN to the NTS contributes to suppression in appetite following acute restraint stress.

#### 3.3.3 NTS and PPG-projecting PVN neurons express *Crh*

RNAscope *in situ* hybridisation revealed that nearly half of NTS-projecting PVN neurons expressed *Crh*, but only a small number were oxytocin-positive, and approximately half of the oxytocin-positive projection neurons also expressed *Crh* mRNA (Figure 5L,M). To confirm that these NTS-projecting PVN^CRH^ neurons provide direct, monosynaptic input to cNTS^PPG^ neurons, we used RNAscope to localize *Crh* mRNA expression in tissue sections from one mouse with monosynaptic retrograde rabies virus labelling of PVN neurons that synapse onto cNTS^PPG^ neurons (Figure 5N,O). Of 59 retrogradely labelled cNTS^PPG^-projecting PVN neurons, the majority (37 neurons, 62.7%) expressed *Crh*.

### 3.4 Exp 4: NTS-projecting PVN^CRH^ neurons are activated following acute restraint

**Figure 6:**
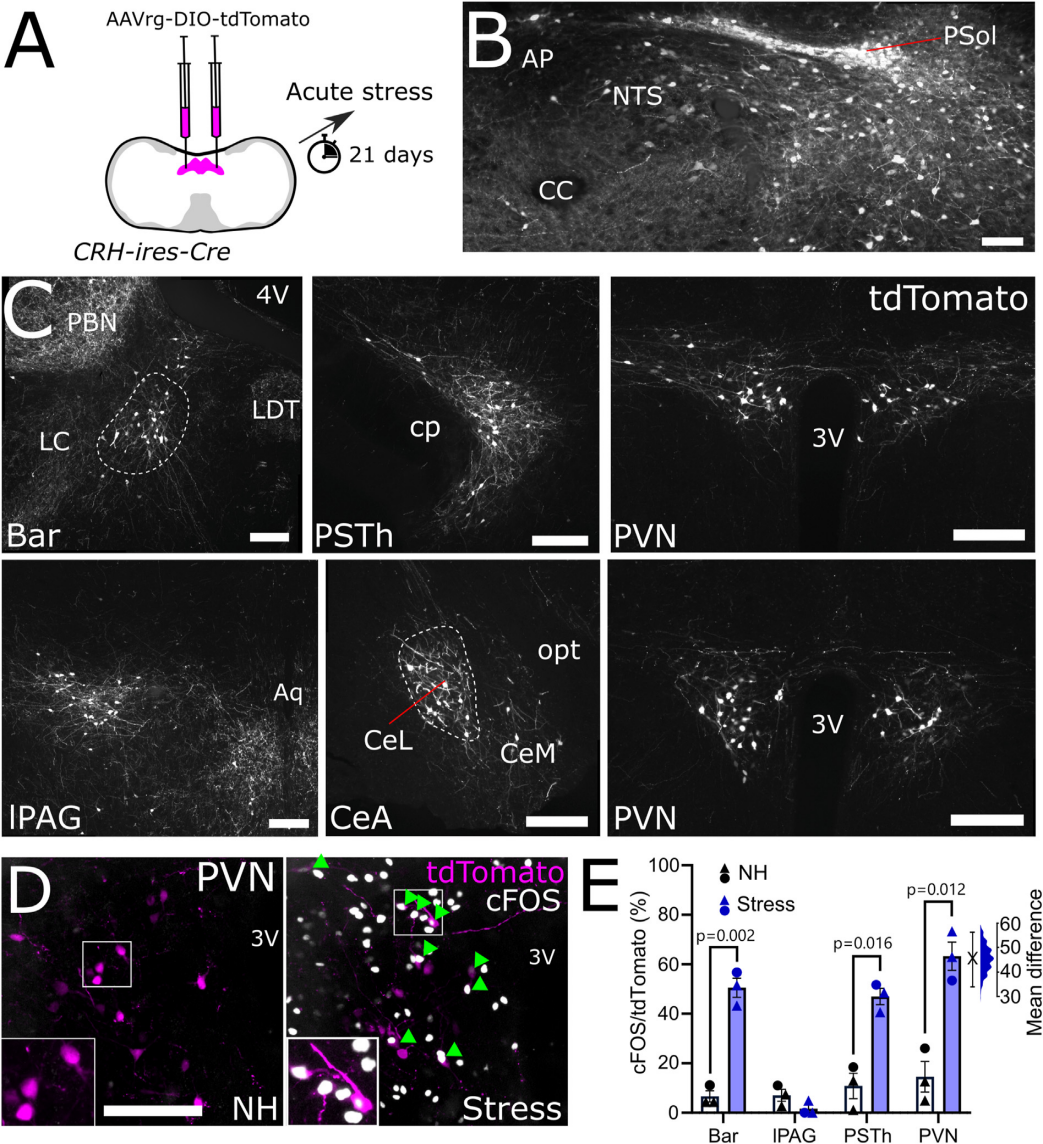
**CRH input to the NTS**. A) Diagram showing injection of retrogradely transported AAV into the NTS of CRH-ires-Cre mice. B) tdTomato immunolabelling in the NTS injection site and surrounding nuclei. C) tdTomato immunolabelling in multiple brain regions, including two levels of the PVN, 21 days after injection into the NTS. D) tdTomato (magenta) and cFOS (grayscale pseudocolour) in the PVN of nonhandled (NH) and stressed mice. E) Percentage of tdTomato-expressing cells in the PVN that were also cFOS-IR in nonhandled mice (NH, N = 2M, 1F) or mice exposed to acute restraint (N = 2M, 1F). Males depicted in triangles and females in circles. Unpaired T-tests with Bonferroni correction: Bar: t = 9.780, df = 4; lPAG: t = 1.866, df = 4; PSTh: t = 5.934, df = 4; PVN: t = 6.423, df = 4. Scale bar: 100 μm; AP: area postrema, PSol: parasolitary nucleus, Bar: Barrington's nucleus, PSTh: parasubthalamic nucleus, lPAG: lateral periaqueductal grey; CeA: central amygdala, CeL: lateral part of the central amygdala, CeM: medial part of the central amygdala, opt: optic tract, 3V: third ventricle.

To confirm our results above and previous findings that PVN^CRH^ neurons project directly to the NTS, male and female CRH-ires-Cre mice were microinjected into the cNTS with AAVrg-CAG-FLEX-tdTomato (Figure 6A). tdTomato-positive cells were scattered in the cNTS injection site and were present in the neighbouring parasolitary nucleus (PSol, Fig 6B). Dense populations of NTS-projecting CRH neurons were identified in Barrington's nucleus, the central amygdala, the lateral periaqueductal gray, the parasubthalamic nucleus (PSTh), and the PVN (Fig 6C). In addition, small numbers of tdTomato-expressing neurons were found in the bed nucleus of the stria terminalis and the lateral hypothalamus (not shown). Multiple populations of NTS-projecting CRH neurons were found to express cFOS following acute restraint as compared to nonhandled mice (Fig 6E), including Barrington's nucleus and the PSTh. The largest effect was seen in cNTS-projecting PVN^CRH^ neurons where restraint stress increased activation by 45.6 %points (95%CI: 34.1, 56.1) compared to non-handled controls (Figure 6D–E), again supporting a role for NTS-projecting PVN^CRH^ neurons in mediating stress responses.

### 3.5 Exp 5: CRH actions on cNTS^PPG^ neurons

**Figure 7:**
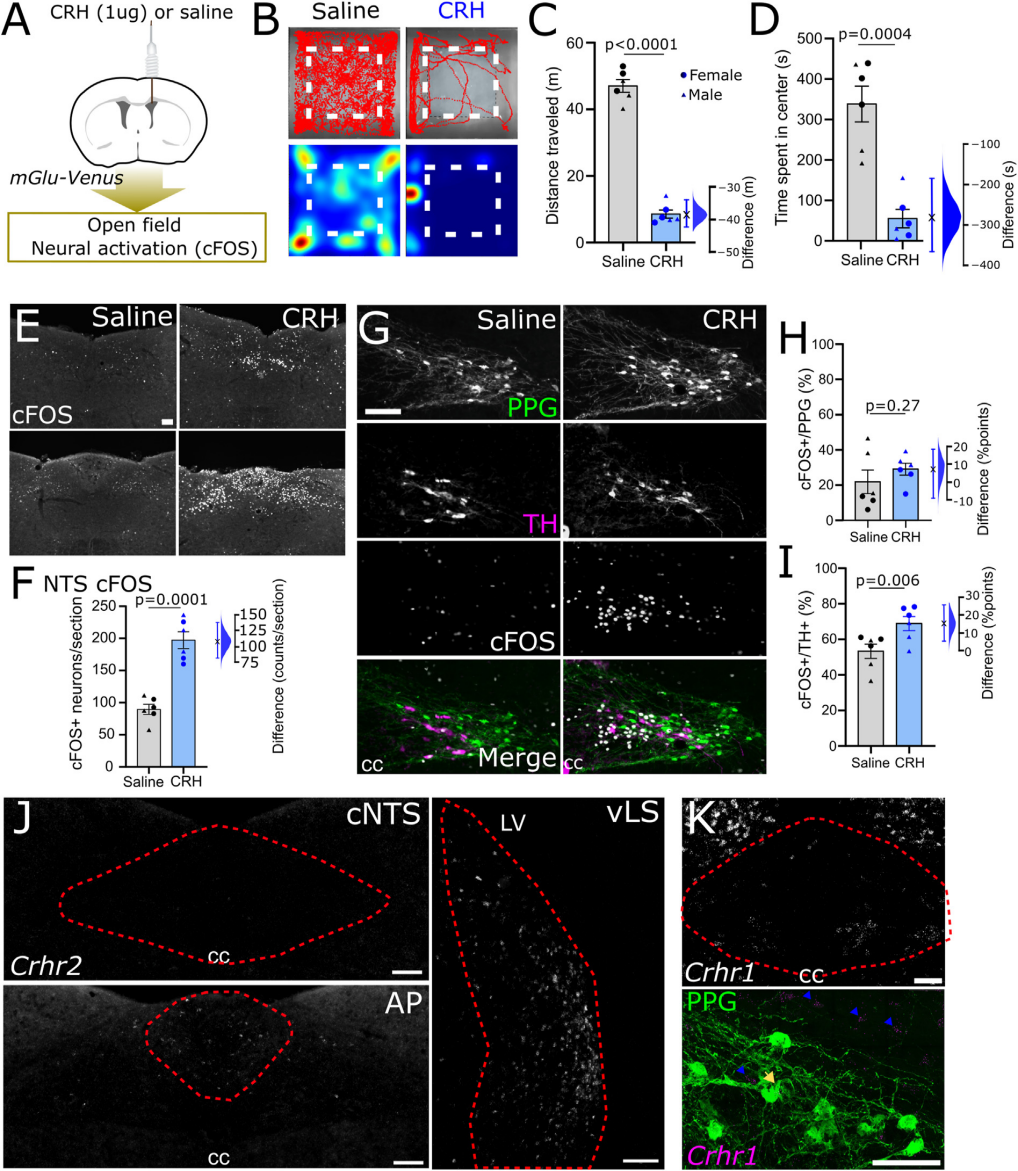
**Actions of CRH on NTS PPG neurons in male and female mice.** A) Diagram of the experimental paradigm for delivery of exogenous CRH (1 μg in 1 μl saline) to the lateral ventricle of mGlu-Venus mice. B) Traces (top) and heatmaps (bottom) recorded in the open field following injection of either saline (left) or CRH (right) into the lateral ventricle. The center of the arena is outlined using a dashed line. C-D) Quantification of the total distance traveled (C) and time spent in the center of the open field (D) of saline and CRH-injected mice. Two-way ANOVA (distance traveled): F(1,8) = 0.95, p = 0.36; main effect of sex: F (1, 8) = 2.536, p = 0.15; main effect of drug: F (1, 8) = 332.5, p < 0.0001. Two-way ANOVA (centre time): F(1, 8) = 1.700, p = 0.23; main effect of sex: F(1, 8) = 0.90, p = 0.37; main effect of drug: F(1, 8) = 34.35, p = 0.0004. E) cFOS IF at two bregma levels of the cNTS after saline (left) or CRH (right). F) Numbers of cFOS-IR neurons per cNTS section in mice treated with either saline or CRH. Two-way drug × sex interaction: F(1,8) = 1.100, p = 0.32; main effect of sex: F(1, 8) = 0.2764, p = 0.61; main effect of drug: F(1,8) = 47.49, p = 0.0001. G) Representative images of immunolabelling for YFP (representing PPG, green), tyrosine hydroxylase (TH, magenta) and cFOS (grayscale) in the cNTS of mGlu-YFP mice injected with saline (left) or CRH (right) into the lateral ventricle. H-J) Percentage of YFP-IR PPG neurons (H) or TH-IR neurons (I) that are also cFOS-IR in the cNTS of mice treated with either saline or CRH. Two-way drug × sex interaction (cNTS^PPG^ neurons): F(1, 8) = 0.5462; p = 0.48; main effect of sex: F(1,8) = 6.986, p = 0.03; main effect of drug: F(1, 8) = 1.428, p = 0.27. Two-way drug × sex interaction (TH): F(1,8) = 0.1571; p = 0.70; main effect of sex: F(1, 8) = 10.58, p = 0.012; main effect of drug: F(1,8) = 14.16, p = 0.0055. J) RNAscope for *Crhr2* in the cNTS (top), area postrema (AP, bottom), and ventral lateral septum (vLS). Entire figure: N = 3F,3M in each group with males depicted in triangles and females in circles. K) FISH for *Crhr1* in the cNTS and neighbouring gracile nucleus (top panel); GFP-IR PPG neurons (green) and FISH for *Crhr1* (magenta) in the cNTS (bottom panel). Blue arrowheads indicate *Crhr1-*positive cells, yellow arrow indicates a single double-labelled neuron. Scale bars = 100 μm. (For interpretation of the references to color in this figure legend, the reader is referred to the Web version of this article.)

Based on our finding that PVN^CRH^ neurons are activated by stress and provide direct input to cNTS^PPG^ neurons, we tested if delivery of exogenous CRH peptide would elicit activation of cNTS^PPG^ neurons. Delivery of a high dose of CRH (1 μg) into the lateral ventricle (Figure 7A) elicited a clear behavioural phenotype in the open field (Figure 7B–D) and a robust increase in cFOS in the cNTS and area postrema (Figure 7E–F). However, CRH delivery had no impact on cNTS^PPG^ neuron cFOS activation (Figure 7G–H), despite increasing the activity of neighbouring noradrenergic (TH-positive) cNTS neurons (Figure 7G,I). Interestingly, there was a main effect of sex on activation of TH neurons with females displaying higher levels of activation in both conditions (Fig 7I). While CRHR2 has been reported to be expressed in the NTS at higher levels than CRHR1 in rats [55], we only detected very low levels of *Crhr2* mRNA within the mouse cNTS (Fig 7J), despite high levels being detected in the ventral lateral septum (Figure 7J right panel) and moderate levels in the area postrema (Figure 7J bottom panel). Contrary to our expectations, *Crhr1* mRNA was expressed to a much higher level than *Crhr2* in the dorsal vagal complex and surrounding areas with the gracile nucleus being most densely labelled (Fig 7K). However, while *Crhr1* was expressed in some areas of the cNTS, we found no evidence for widespread expression of *Crhr1* by cNTS^PPG^ neurons (Fig 7K). Our collective findings suggest that while PVN^CRH^ neurons provide input to the cNTS, including synaptic input to cNTS^PPG^ neurons, and despite evidence that NTS-projecting PVN^CRH^ neurons are activated following restraint stress, CRH receptor signalling may not be responsible for activation of cNTS^PPG^ neurons in response to this psychogenic stressor.

## 4 Discussion

We report that cNTS^GLP−1/PPG^ neuronal activation can elicit anxiety-like behaviours in mice in a sex-dependent manner. We also show that activation of these neurons following acute stress relies on input from the PVN, which contributes to stress-induced suppression in feeding. Our finding that cNTS^PPG^ neural activation suppresses feeding partly by decreasing the size of the first meal (with no impact on meal frequency) is consistent with acceleration of satiation, as reported previously by Brierley et al. [21]. However, an increase in the latency to begin feeding together with a decrease in first meal size can also be indicative of behavioural suppression, possibly associated with negative emotional state [14]. Indeed, in addition to a well-established hypophagic role of central GLP-1, solid evidence supports an anxiogenic role in male and female rats, in which anxiety-like behaviours increase upon GLP-1 receptor stimulation in the central amygdala [33] or supramammillary nucleus [37], and decrease following GLP-1 receptor knockdown in the bed nucleus of the stria terminalis [36]. A previous study failed to observe any anxiogenic effects of chemogenetic activation of cNTS^PPG^ neurons when mice were tested in the elevated plus maze or open field [23]. However, that study only tested male mice (Ronald Gaykema, personal communication) and only used behavioural assays dependent on exploratory behaviour, which is reduced by cNTS^PPG^ neuron activation (this report and [23]). Our finding that cNTS^PPG^ neural activation moderately increases anxiety-like behaviour in the open field assay in female (but not male) mice supports a sex-dependent anxiogenic effect of GLP-1/PPG neurons in this behavioural assay. Our new findings using the acoustic startle test indicate that both male and female mice respond to cNTS^PPG^ neuron activation with an increased startle response indicative of increased arousal and/or vigilance, both of which are recognized components of anxiety-like behaviour in mice, rats, and humans [56].

### 4.1 Potential sex differences

Previous studies, including our own, have demonstrated robust feeding suppression in mice following optogenetic or chemogenetic activation of cNTS^PPG^ neurons. However, these studies reported data collected only in males [23,25,35], or were underpowered to reveal potential sex differences [21,22,24]. Here we document that after chemogenetic activation of cNTS^PPG^ neurons, female mice displayed hypophagia for 6 h, whereas the effect in males was more transient. In both sexes, food intake and body weight normalized within 24 h after CNO treatment. Previous studies reported variable hypophagia effect durations in mixed-sex groups of mice after acute chemogenetic activation of cNTS^PPG^ neurons, with results indicating either no persistent effect on 24h food intake and body weight (this report and [24]) or a sustained effect on cumulative food intake and body weight lasting up to 48 h after a single injection of CNO [21]. The reason for this discrepancy is unclear but may be due to differences in functional expression of the hM3Dq construct, including whether the population of cNTS^PPG^ neurons in the adjacent intermediate reticular nucleus is transduced, and resulting variability in the level of activation as well as the relative numbers of male and female mice used in the study.

Based on the evidence presented here and by Gaykema et al. [23], we propose that cNTS^PPG^ neural activation contributes to hypophagia and other behavioural indices of anxiety-like behaviour in a manner that is partially sex- and assay-dependent. In this regard, sex differences in other behavioural responses to GLP-1 receptor stimulation and inhibition have been reported in rats [37]. Collectively, these findings highlight the importance of including experimental subjects of both sexes in a variety of assays and support the hypothesis that the central GLP-1 system modulates behaviour in a sex-dependent manner.

### 4.2 Functional and anatomical evidence for a PVN-NTS pathway inhibiting feeding in response to stress

It has been reported that hindbrain 5-HT contributes to stress-induced activation of GLP-1 neurons in rats [11]. Here we show that NTS-projecting PVN neurons are another necessary source of input for the ability of restraint stress to activate GLP-1/PPG and other cNTS neurons in male wildtype mice. Our new finding is reminiscent of the demonstrated contribution of descending PVN axonal projections in stress-induced recruitment of noradrenergic NTS neurons in rats [57,58]. Importantly, the axon collaterals of NTS-projecting PVN neurons targeted additional brain regions that may contribute to activation of cNTS^PPG^ neurons. In this regard, there is evidence that PVN projections to the mesencephalic and pontine periaqueductal gray and parabrachial nuclei contribute to the feeding-suppressive effects of PVN stimulation [59]. However, we observed very few labelled axon collaterals in those two regions arising from NTS-projecting PVN neurons. Interestingly, in addition to attenuating stress-induced hypophagia, acute inhibition of NTS-projecting PVN neurons increased dark-onset food intake in non-stressed male mice. This result was surprising, given previous reports that inhibition of PVN-derived axon terminals within the NTS failed to increase daily feeding in *ad libitum*-fed male rats [60], failed to increase intake in refed male rats after overnight food restriction [60] and did not increase intake in predominantly male mice in the early hours of the light phase [59]. The reason for the discrepancy is unclear although timing of intervention (light phase in previous studies versus dark onset in the present study) and the concomitant activation of axon collaterals targeting other regions in our study may contribute. It is important to note that our examination of NTS-projecting PVN neurons was restricted to feeding behaviour in male mice due to limited availability of experimental animals during the COVID-19 pandemic, followed by relocation of the lead author. Sex differences in the hypophagic response to stress have been reported in previous studies, but with conflicting results. One study reported that females were less susceptible to hypophagia following acute restraint [61], while another reported that males were less susceptible [62]. It is likely that age, housing conditions and the precise stress protocol utilised also impact stress-induced hypophagia [63,64]. Future studies building on the present results should explore the role of NTS-projecting PVN neurons in females, and should address whether this descending neural pathway contributes to other stress-related behaviours.

### 4.3 The role of CRH in stress-induced activation of cNTS^PPG^ neurons

Considering our finding that approximately half of NTS-projecting PVN neurons express *Crh* mRNA, consistent with previous reports in rats [65,66], it is possible that chemogenetic inhibition of NTS-projecting PVN neurons also attenuated adrenocorticotropic hormone (ACTH) and corticosterone release following acute stress. We did not measure plasma levels of these stress hormones, but the lack of axon collaterals in the median eminence originating from NTS-projecting PVN neurons is consistent with other evidence that neuroendocrine PVN neurons comprise a population that is distinct from brainstem-projecting PVN neurons [[67], [68], [69]]. The existence of a monosynaptic circuit from the PVN to cNTS^PPG^ neurons shown here and previously [10], coupled with evidence that cNTS^PPG^-projecting PVN neurons and NTS-projecting PVN^CRH^ neurons are activated after acute stress, support the view that a direct pathway from PVN to NTS contributes to stress-induced activation of cNTS^GLP−1/PPG^ neurons and that PVN^CRH^ neurons may form part of this pathway. PVN^CRH^ neurons are well-established drivers of neuroendocrine and behavioural responses to stress [3,70,71]. NTS-projecting PVN^CRH^ neurons have been demonstrated to increase blood pressure [72], although the role of these NTS-projecting CRH neurons in response to stress has not previously been explored.

Interestingly, CRH delivered directly to the lateral ventricle did not activate cNTS^PPG^ neurons despite clear behavioural responses to the treatment and activation of cNTS^TH^ neurons. In addition, we found no evidence that cNTS^PPG^ neurons express CRH receptors. Importantly, CRHR2 has been found in rats to be expressed almost exclusively on fibers, not by cell bodies, in the cNTS [65,73] and CRHR2 is abundantly expressed in the nodose ganglion in mice [74], where it's been shown to modulate mechanosensitivity [75]. It is therefore plausible, that any effects of CRH in the cNTS is primarily via presynaptic modulation of vagal afferent fibres.

Collectively this suggests that while input from the PVN is necessary for restraint stress-induced activation of cNTS^PPG^ neurons, CRH acting *directly* on cNTS^PPG^ may not be the main driver of this response. Future studies should explore whether glutamate is the neurotransmitter responsible for cNTS^PPG^ neuronal activation, given its co-expression in PVN^CRH^ neurons [76], and should investigate the role of presynaptic CRHR2. As an alternative explanation, stress-induced activation of cNTS^PPG^ neurons may depend on CRH signalling, but requiring non-CRH converging inputs for activation. This could explain why exogenous CRH delivered centrally in the absence of a concurrent stressor had no impact on cNTS^PPG^ neuron cFOS expression.

### 4.4 Conclusions

In rodents, acute stressors increase vigilance and arousal, suppress food intake, and reduce exploratory and approach behaviours through engagement of complex neural circuits. Our findings highlight a hypothalamic-brainstem pathway from CRH neurons in the PVN to GLP-1/PPG neurons in the cNTS in the modulation of behavioural responses to stress. We demonstrate that chemogenetic activation of GLP-1-producing PPG neurons in the cNTS of mice suppresses food intake and increases anxiety-like responses in a partially sex-dependent manner. These findings along with previously published studies using rats and mice support the view that cNTS^GLP−1/PPG^ neurons are an integral component of neural circuits that modulate physiological and behavioural responses to acute stress, including increased heart rate [27,35], reduced feeding (this report and [24]), and increased anxiety-like behaviour (this report and [36,37]). We further demonstrate that cNTS^PPG^-projecting PVN neurons are activated by restraint stress, and that NTS-projecting PVN neurons are necessary for the ability of restraint stress to activate PPG and other cNTS neurons in mice. The chemical phenotype of those additional populations is unknown, but they likely include catecholaminergic A2 neurons [53,57,58].

We discovered that chemogenetic inhibition of NTS-projecting PVN neurons, approximately half of which express the stress neuropeptide CRH, increases food intake under baseline conditions and attenuates stress-induced hypophagia. Finally, while NTS-projecting PVN^CRH^ neurons were activated following acute stress, exogenous CRH delivered to the lateral ventricle was unable to activate cNTS^PPG^ neurons. These novel findings highlight a descending pathway from the hypothalamus to the cNTS within a broader neural system that orchestrates behavioural stress responses.

## CRediT authorship contribution statement

**Marie K. Holt:** Writing – review & editing, Writing – original draft, Visualization, Methodology, Investigation, Funding acquisition, Formal analysis, Conceptualization. **Natalia Valderrama:** Writing – original draft, Investigation, Funding acquisition, Formal analysis. **Maria J. Polanco:** Investigation. **Imogen Hayter:** Investigation. **Ellena G. Badenoch:** Investigation, Writing – review & editing. **Stefan Trapp:** Writing – review & editing, Resources, Funding acquisition. **Linda Rinaman:** Writing – review & editing, Writing – original draft, Funding acquisition, Conceptualization.

## Declaration of competing interest

The authors declare that they have no known competing financial interests or personal relationships that could have appeared to influence the work reported in this paper.

## Data Availability

Data will be made available on request.
